# Evolution of ferroelectricity in ultrathin PbTiO_3_ films as revealed by electric double layer gating

**DOI:** 10.1038/s41598-020-67580-8

**Published:** 2020-07-02

**Authors:** Ryutaro Nishino, Takahiro C. Fujita, Fumitaka Kagawa, Masashi Kawasaki

**Affiliations:** 1 Department of Applied Physics and Quantum-Phase Electronics Center, University of Tokyo, Bunkyo, Tokyo 113-8656 Japan; 2 RIKEN Center for Emergent Matter Science (CMES), Wako, Saitama 351-0198 Japan

**Keywords:** Condensed-matter physics, Techniques and instrumentation, Materials science, Physics

## Abstract

Ferroelectricity in ultrathin films is destabilized by depolarization field, which leads to the reduction of spontaneous polarization or domain formation. Here, thickness dependence of remnant polarization in PbTiO_3_ films is electrically revealed down to 2.6 nm by controlling the polarization direction with employing an electric double layer gating technique to suppress leakage current in ultrathin films. The remnant polarization for a 17 nm-thick film is similar to bulk value ~ 60 μC cm^−2^ and reduces to ~ 20 μC cm^−2^ for a 2.6 nm-thick film, whereas robust ferroelectricity is clearly observed in such ultrathin films. In-situ X-ray diffraction measurements under an external electric field reveal that the reduced tetragonality in ultrathin films is mostly recovered by cancelling out the depolarization field. Electric double layer gating technique is an excellent way for exploring physical properties in ultrathin ferroelectric films.

## Introduction

The evolution of electric polarization with reduced film thickness has been one of the important issues in the field of thin film ferroelectrics^1,2^. Depolarization field, which is caused by imperfect screening of spontaneous polarization^3,4^, crucially destabilizes ferroelectricity, leading to the reduction of polarization^5^ or the pinned polydomain state^6^ in the ultrathin film regime. For instance, theoretical calculation^4^ has predicted that critical thickness, below which the ferroelectricity disappears, is 6-unit cells in BaTiO_3_, and the reduction of polarization was confirmed experimentally^7^. In the case of PbTiO_3_, the tetragonality (the ratio of *c*- to *a*-axes lattice constants in tetragonal crystals) is suppressed with decreasing thickness, indirectly inferring the suppression of polarization^8^. Instead of the reduction in polarization, creation of a pinned polydomain is another solution to mitigate the depolarization field, which is also experimentally confirmed in PbZr_0.2_Ti_0.8_O_3_ (PZT)^6^ and BiFeO_3_^9^ thin films.

While ferroelectricity in ultrathin films has been investigated by a scanning probe technique such as a piezoelectric force microscopy^10^ (PFM) or structural analyses with an X-ray diffraction^11^ and an electron microscopy^12^, the traditional electrical measurement in a capacitor structure has been scarcely reported due to large leakage current in ultrathin films. This problem makes characterizations of basic ferroelectric properties such as remnant polarization and coercive field unreliable even though they are of great importance to clarify the evolution of ferroelectricity in low dimensionality. Experimentally, polarization hysteresis loop was measured for 5 nm-thick BaTiO_3_ films^5^ and for 19 nm-thick PbTiO_3_ films^13^, while leakage current prohibited the measurement below those thicknesses. Recently, there have been reported examples of polarization reversal^14,15^ and measurement^16^ with an electric double layer (EDL) formed at an interface between ionic liquid (IL) and a ferroelectric film. In this technique, mobile ions in the IL are accumulated on the ferroelectric surface by applying an external electric field and then the polarization is reversed to screen the charge of these ions. Since IL is ionically conductive but electrically insulative, this technique has the great advantage of suppressing leakage current and evaluating polarization in ultrathin films compared to the conventional measurement with a capacitor structure.

Here, we report the thickness-dependent ferroelectric properties in PbTiO_3_ films measured with the EDL structure. The in-plane lattice constant of PbTiO_3_ (*a* = 3.90 Å)^17^ is close to that of SrTiO_3_ substrate (*a* = 3.905 Å). This very small lattice mismatch enables coherent growth in a wide range of thickness without strain relaxation, which otherwise strongly affects ferroelectric properties such as tetragonality, coercive field^18^ and polarization^19^. Generally, such strain relaxation makes the thickness-dependent behavior complicated. Therefore, PbTiO_3_/SrTiO_3_ is a suitable system for exploring thickness-dependent properties. We demonstrate that the EDL gating technique enables us to control polarization direction and to measure remnant polarization of ultrathin PbTiO_3_ films down to 2.6 nm. PbTiO_3_ retains ferroelectricity at 2.6 nm-thick, although the remnant polarization is reduced to 20 from 60 μC cm^−2^ for thicker films. The reduction in the remnant polarization comes from not only the decrease of the spontaneous polarization but also the polarization relaxation (i.e. the formation of a polydomain state) to suppress the depolarization field. Polarization control with the EDL gating also enables an in-situ XRD measurement of the tetragonality under an external electric field application for cancelling out the depolarization field. The tetragonality in thinner films, which is suppressed by the depolarization field, recovers by the applied electric field but does not reach the value measured in a thick sample, implying that unrevealed factors suppress ferroelectricity in ultrathin films in addition to the depolarization field.

## Results and discussion

**Figure 1:**
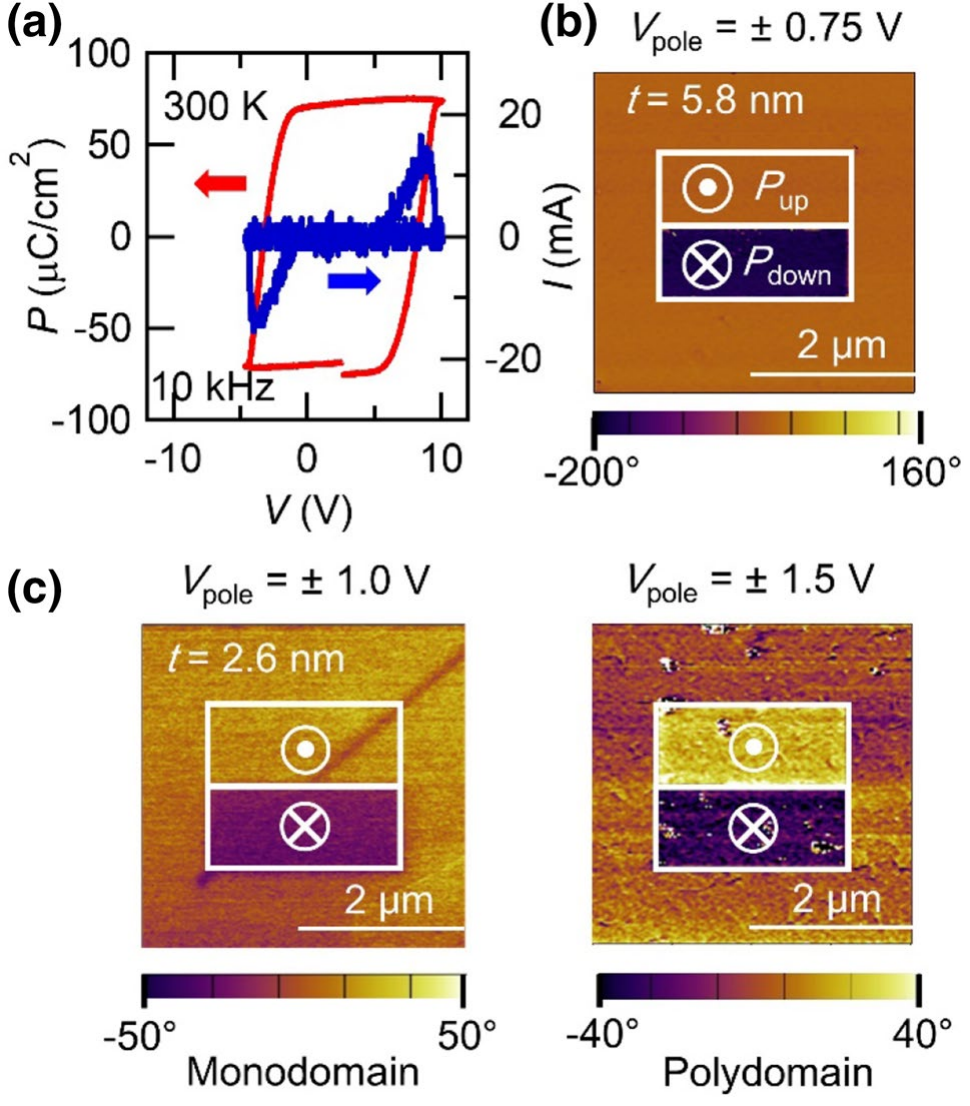
Ferroelectricity and domain structures in PbTiO_3_ films. (**a**) Polarization (*P*) voltage (*V*) loop (red, left axis) and displacement current (blue, right axis) measured at 10 kHz and 300 K for a Au/Ni/PbTiO_3_ (500 nm)/SrRuO_3_ capacitor structure. Au/Ni contact were patterned to a diameter of 110 μm. Phase contrast images measured by a piezoelectric force microscope for (**b**) 5.8 and (**c**) 2.6 nm-thick PbTiO_3_ films. Before the measurement, negative and positive biases (*V*_pole_) were applied in upper and lower rectangle regions to induce upward (pointing away from the substrate: *P*_up_) and downward (pointing toward the substrate: *P*_down_) polarization, respectively. Monodomain and polydomain region are mixed in one sample for 2.6 nm-thick film.

PbTiO_3_ thin films were grown on atomically smooth SrRuO_3_ (~ 5 nm-thick) buffered (001) SrTiO_3_ substrates by pulsed laser deposition. The details of growth are already reported elsewhere^20^. Figure 1a shows the polarization-voltage hysteresis loop measured for a capacitor structure of Au/Ni/PbTiO_3_(500 nm)/SrRuO_3_. The remnant polarization and coercive field in this capacitor appeared to be 70 μC cm^−2^ and 110 kV cm^−1^, respectively, being similar to those in previous reports^13,20^. The asymmetric hysteresis loop may be caused by the difference of workfunctions between top and bottom electrodes^20,21^.

To confirm ferroelectricity and domain structures in the thinner PbTiO_3_ films, we performed a PFM measurement. The phase contrast of the piezoresponse for 5.8 and 2.6 nm-thick PbTiO_3_ films are shown in Fig. 1b, c, respectively (see Supplementary Fig. S1 for the PFM amplitude images). Prior to the measurement, negative and positive biases were applied to polarize the upper and lower rectangles, respectively. Here, negative and positive voltages induce upward (pointing away from the substrate) and downward (pointing toward the substrate) polarizations, respectively. For the 5.8 nm-thick sample, the PFM phase of the upper rectangle shows the same contrast to that of the as-grown region (Fig. 1b), indicating that the 5.8 nm-thick PbTiO_3_ thin film is monodomain with upward polarization at as-grown state. For the 2.6 nm-thick sample, phase difference between upper and lower rectangles is below 180° (see Supplementary Fig. S1). In ultrathin films, piezoresponse is so small that an instrument background cannot be ignored, which results in a smaller phase shift contrast than 180°^22^. We also conducted a Kelvin probe force microscopy measurement to investigate an effect of injected charges around the poled regions (see Supplementary Fig. S2). The surface potential of the poled regions shows an opposite sign to the direction of remnant polarization and it is stable for long time, indicating that remnant polarization prevents the injected surface charges from diffusing. Therefore, we conclude that the polarization in the poled regions is switched by an electric field. In contrast to the 5.8 nm-thick sample, the 2.6 nm-thick sample gives a different contrast depending on the sample position on the substrate. One is monodomain with upward polarization (left Fig. 1c) and the other shows the possibility of a polydomain state (right Fig. 1c). The PFM phase of the as-grown region shows the mean value between the upper and lower rectangles (see Supplementary Fig. S1(c) and (d)), indicating the presence of upward and downward polarized domains. Generally it is difficult to resolve each domain because domain size decreases with reducing film thickness and expected domain size is few nm order in ultrathin films^23^, which is smaller than resolution of our measurements. However, similar indistinguishable phase contrast was reported in polydomain PbTiO_3_ films^24^. Therefore, we speculate that 2.6 nm-thick samples take polydomain state at some position. The polydomain configuration may arise to suppress the increasing depolarization field with decreasing thickness as exemplified by previously reported PbTiO_3_/La_0.67_Sr_0.33_MnO_3_ heterostructures^25^. Since the upper and lower rectangles show the different phase contrast, the polydomain region can be polarized by an electric field, indicating that the finite remnant polarization remains down to the 2.6 nm-thick PbTiO_3_ film.

**Figure 2:**
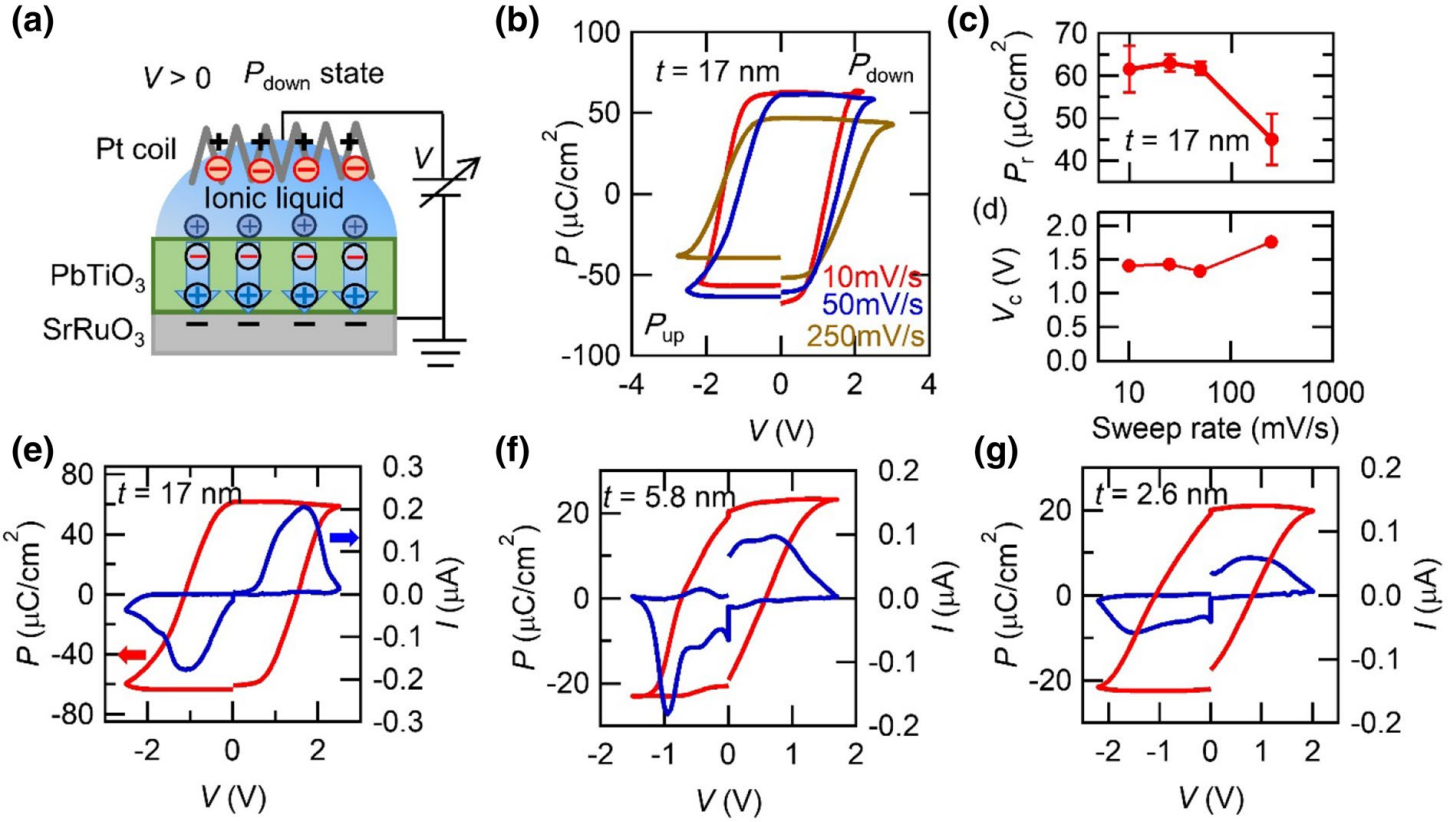
Polarization measurement in PbTiO_3_ films. (**a**) Schematic of the device for polarization measurement with employing an electric double layer gating. Ions and polarization alignment are described for positive voltage. (**b**) Polarization (*P*) voltage (*V*) loop deduced by the positive-up negative-down (PUND) method for 17 nm-thick PbTiO_3_ film measured at various sweep rates. Positive and negative voltage stabilize downward (*P*_down_ state) and upward (*P*_up_ state) polarization, respectively. (**c**) Remnant polarization (*P*_r_) and (**d**) coercive voltage (*V*_C_) in 17 nm-thick film as a function of sweep rate. *P*–*V* loop (red, left axis) and displacement current (blue, right axis) for (**e**) 17, (**f**) 5.8, and (**g**) 2.6 nm-thick PbTiO_3_ films deduced by the PUND method. *V* is scanned at 50 mV s^−1^.

We then measured remnant polarization (*P*_r_) and coercive voltage (*V*_C_) in PbTiO_3_ films with the EDL structure composed of Pt coil/IL/PbTiO_3_/SrRuO_3_ as shown in Fig. 2a. We employed the positive-up negative-down (PUND) method to obtain the accurate remnant polarization^16^. This method can extract pure displacement current component by subtracting other components coming from leakage current, dielectric polarization and polarization relaxation. We performed X-ray diffraction measurement around PbTiO_3_ (001) peaks and confirmed that peak intensity and position stayed almost unchanged after PUND measurements. Therefore, electrochemical reaction, such as oxygen migration, induced by the EDL gating plays minor effect, if any, for this measurement. We define *V*_C_ as a point at which polarization is zero in a hysteresis loop obtained by this method. Since motion of ions in IL is slow^26^, the ions cannot perfectly follow an applied voltage under a high sweeping rate and thus we first examined the sweep rate dependence of the *P*_r_ and *V*_C_. Figure 2b shows *P* -*V* hysteresis loop in a 17 nm-thick sample measured under various sweep rates. At a rate of 250 mV s^−1^, the *P*_r_ significantly decreases and the hysteresis loop expands, indicating that the ions cannot fully respond to the applied voltage at this sweep rate. The measured *P*_r_ and *V*_C_ as a function of sweep rate are summarized in Fig. 2c, d, respectively. Provided that the sweep rate below 50 mV s^−1^ gives saturation in the *P*_r_ and *V*_C_, all the measurements were performed at a rate of 50 mV s^−1^ hereafter.

Figure 2e–g are the measured hysteresis loops for the samples with various thicknesses. In the 17 nm-thick sample, the *P*_r_ is around 62 μC cm^−2^ which is close to that of the thick film obtained by the conventional capacitor structure (*P*_r_ = 70 μC cm^−2^, Fig. 1a). On the other hand, the *P*_r_ is reduced to ~ 20 μC cm^−2^ in the 5.8 and 2.6 nm-thick samples. This reduction of the *P*_r_ mainly comes from the enhancement of depolarization field with reducing film thickness, which will be discussed in more detail later. Here, we note that a PbTiO_3_ film as thin as 2.6 nm shows clear reversible polarization by the electrical measurement. This demonstrates that the EDL gating is capable of switching and evaluating polarization in ultrathin ferroelectric films.

**Figure 3:**
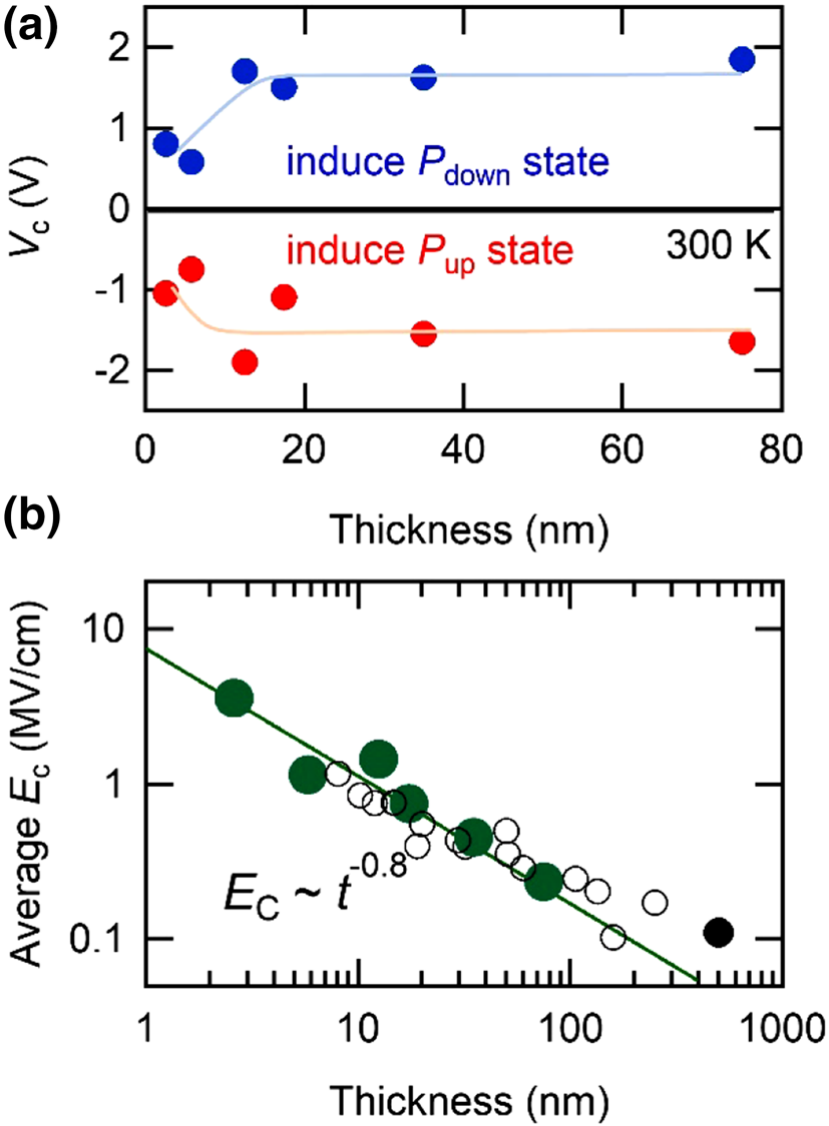
Thickness dependence of Coercive voltage and field. (**a**) Coercive voltage (*V*_C_) as a function of film thickness. Positive and negative voltage induce downward (*P*_down_ state) and upward (*P*_up_ state) polarization, respectively. Blue and red lines are guide to eye. (**b**) Relationship between coercive field (*E*_C_) and the thickness of PbTiO_3_ films. Solid green and black circles are those measured by the electric double layer structure or the conventional capacitor structure in this study, respectively. Open circles are those for Pb(Zr,Ti)O_3_ films reported by several other groups^13,18,27^. The solid green line is a fitting to *E*_C_ ~ *t*^−0.8^ relation.

The thickness dependence of *V*_C_ is summarized in Fig. 3a. The *V*_C_ is almost constant in thicker films and slightly reduced below 12 nm. In other word, coercive field (*E*_C_ = *V*_C_/*t*, *t*: thickness) is not constant as a function of the film thickness as shown in Fig. 3b. The reported values for Pb(Zr,Ti)O_3_ films are also plotted, which were measured with conventional capacitor structures^13,18,27^. The measured coercive field increases drastically as the thickness decreases and the *E*_C_ can be fitted by *E*_C_ ∝ *t*^−0.8^. It should be noteworthy that the *E*_C_ measured with the EDL structure shows the similar values and scaling behavior to those of the previous studies even though the device structure is different. Since IL contacts on a ferroelectric surface in the EDL structure, an equivalent electric circuit is considered as a series capacitance of the EDL and ferroelectrics. In such a case, an applied voltage is divided into the two parts. Hence, the applied voltage is not the voltage that the ferroelectric layer feels. However, the voltage applied to EDL should be zero at *V*c, where upward and downward polarization domains have the same area, because the macroscopic polarization is zero, resulting in the similar value of *E*_c_ measured by both structures. There are several models explaining the thickness dependence of the *E*_C_. For instance, the domain nucleation model^28^ predicts a semiempirical scaling law, where *E*_C_ ∝ *t*^−2/3^. Some studies^29,30^ consider the effects of interfacial dead layer between the ferroelectric layer and electrodes. In this case, *E*_C_ is apparently enhanced with reducing thickness and scaled as *E*_C_ ∝ *t*^−1^. Although the origin of this scaling behavior is still under debate, these observations imply its universality regardless of measurement techniques.

**Figure 4:**
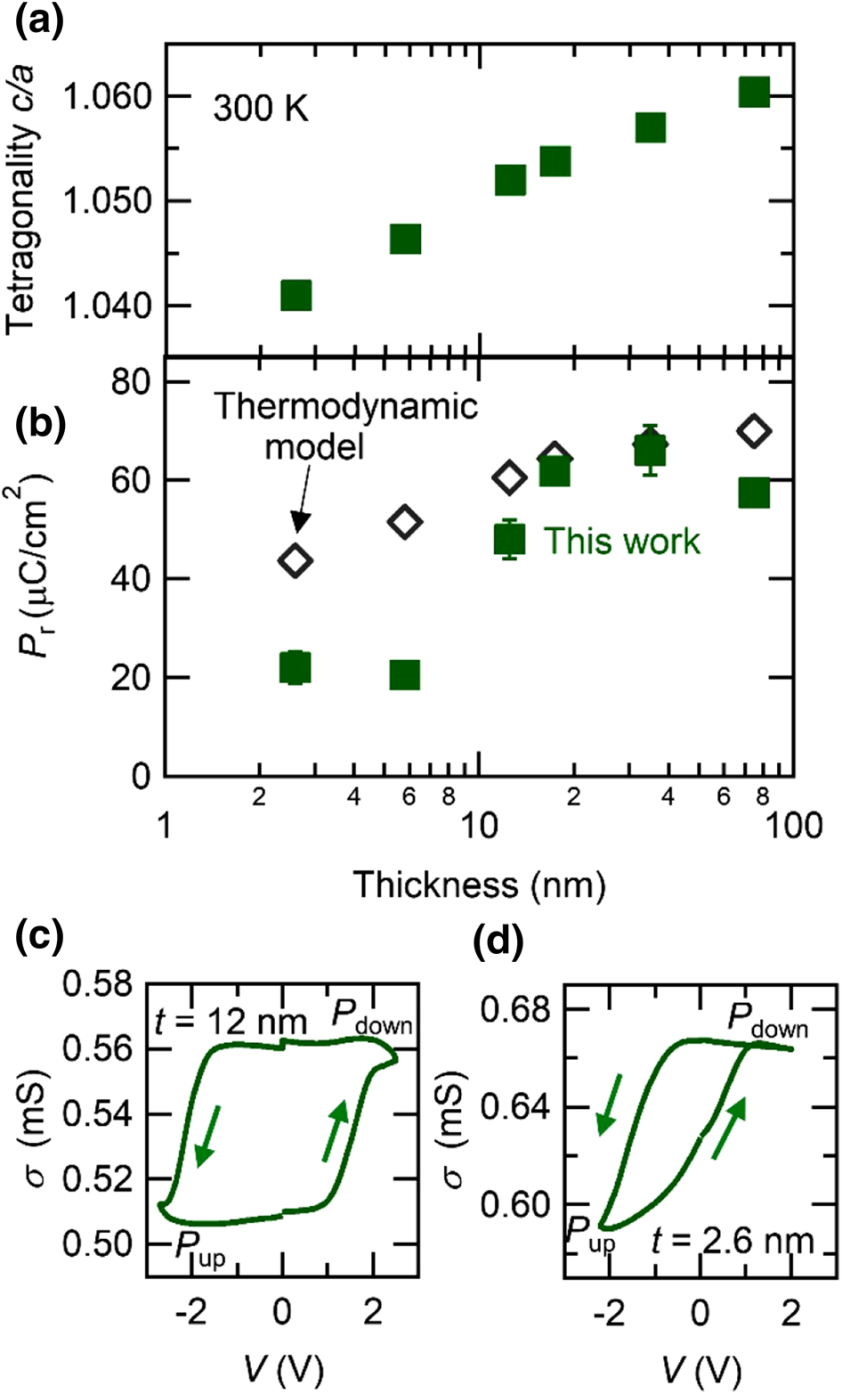
Evolution of ferroelectricity in PbTiO_3_ films. (**a**) Evolution of the measured tetragonality *c/a* (green square) as a function of film thickness. (**b**) Thickness dependence of the remnant polarization (*P*_r_). Green square is the measured values with employing electric double layer structure. Black diamonds show the polarization calculated from the measured *c*-axis lattice constant with thermodynamic model given in Eq. (1). Two terminal conductance (σ) of SrRuO_3_ bottom electrodes for (**c**) 12 and (**d**) 2.6 nm-thick PbTiO_3_ films while voltage (*V*) is scanned at 50 mV s^−1^. Positive and negative voltage stabilize downward (*P*_down_ state) and upward (*P*_up_ state) polarization, respectively.

Next, we examined the evolution of tetragonality and polarization with thickness. Figure 4a shows the tetragonality (*c*/*a*) of PbTiO_3_ films as a function of film thickness. The measured tetragonality exhibits a monotonical decrease as the film thickness reduces, agreeing with the trend of reduced remnant polarization shown in Fig. 2e–g. Figure 4b shows the thickness dependence of the measured remnant polarization. To relate the polarization to the tetragonality, we adopted the thermodynamic model^31^, which dictates spontaneous polarization purely from lattice constant under uniform strain through entire thickness^13,32^ described by

1 $${P}^{2}=\frac{1}{{Q}_{11}-\frac{2{s}_{12}}{{s}_{11}+{s}_{12}}{Q}_{12}}\left[\frac{c}{{a}_{0}}-(1+\frac{{2s}_{12}}{{s}_{11}+{s}_{12}}{u}_{0})\right]$$

where *a*_0_ is the cubic lattice constant of PbTiO_3_, *u*_0_ is the in-plane strain imposed by the SrTiO_3_ substrates described by u_0_ = (*a*_sub_ − *a*_0_)/*a*_0_ (*a*_sub_ : lattice constant of SrTiO_3_), *s*_11_ and *s*_12_ are the elastic compliance coefficients, and *Q*_11_ and *Q*_12_ are the electrostrictive constants. Using *a*_0_ = 3.96 Å, *a*_sub_ = 3.90 Å, *Q*_11_ = 8.9 × 10^−2^ m^4^ C^−2^, *Q*_12_ =  − 2.6 × 10^−2^ m^4^ C^−2^, from ref^31^ and *s*_11_ = 8.0 × 10^−12^ m^3^ F C^−2^, *s*_12_ = − 2.5 × 10^−12^ m^3^ F C^−2^ from ref^32^, the polarization calculated by Eq. (1) with the measured *c*-axis lattice constant is shown as open diamond in Fig. 4b. While the measured remnant polarization is in good agreement with the calculated value for the films thicker than 17 nm, it deviates from the calculation for the thinner films. The remnant polarization substantially decreases below 12 nm and retains almost constant values below 5.8 nm.

To have insight into the origin of this deviation, we consider the possibility of polarization relaxation in the thinner films. In this study, we employed the PUND method to evaluate the remnant polarization, which is a surface average over both upward and downward polarization domains. Hence, the remnant polarization is reduced by polarization relaxation and becomes less than the spontaneous polarization, resulting in the deviation from the calculated spontaneous polarization by Eq. (1). To test this scenario, we measured a conductance (*σ*) of SrRuO_3_ bottom electrode as a function of an applied gate voltage. Due to carrier density modulation induced by ferroelectric field effect, SrRuO_3_ changes its conductance depending on average over both polarization states of PbTiO_3_. Therefore, we can indirectly detect polarization relaxation through these measurements. Figure 4c, d show the conductance change of SrRuO_3_ layer with 12 and 2.6 nm-thick PbTiO_3_, respectively. In both samples, the SrRuO_3_ layer shows high (low) conductance at a positive (negative) voltage, which is consistent with a ferroelectric field effect reported for SrRuO_3_^33^.

In the case of 12 nm-thick sample, the polarization relaxation is negligible since there is no significant difference of the conductance between at 0 V and at saturation voltage. On the other hand, it is clearly observed that the conductance of SrRuO_3_ layer decays from the saturation voltage to 0 V in the 2.6 nm-thick sample. Despite the absence of discernable polarization relaxation for the 12 nm-thick sample, the measured polarization is different from the calculated value. This deviation thus means that the spontaneous polarization reduces more rapidly than that predicted by Eq. (1). This equation dose not explicitly include the energy associated to the coupling between the depolarization field and the polarization, resulting in the deviation from the calculation in thinner film region. In much thinner samples, as in 2.6-nm thick one, polarization relaxation occurs in addition to the reduction of spontaneous polarization. From these results, we find that when PbTiO_3_ films become thinner from a sufficiently thick (> 17 nm) regime, initially the spontaneous polarization is reduced preserving monodomain state (17–12 nm), and then, the films partially take polydomain state (< ~ 12 nm) to mitigate the depolarization field. It might be worthy of note that the thickness limit for the polydomain limit estimated by the polarization measurements is seemingly inconsistent with the PFM measurements. This can be explained by considering internal fields in ferroelectrics such as built-in potential and depolarization field, which are very sensitive to surface and interface properties. In the polarization measurements, PbTiO_3_ surface is in contact with ionic liquid, while it is exposed to the air during the PFM measurements. Therefore, PbTiO_3_ could feel different internal fields between these measurements even though samples have the same film thickness, which may result in the apparently different thickness limit.

**Figure 5:**
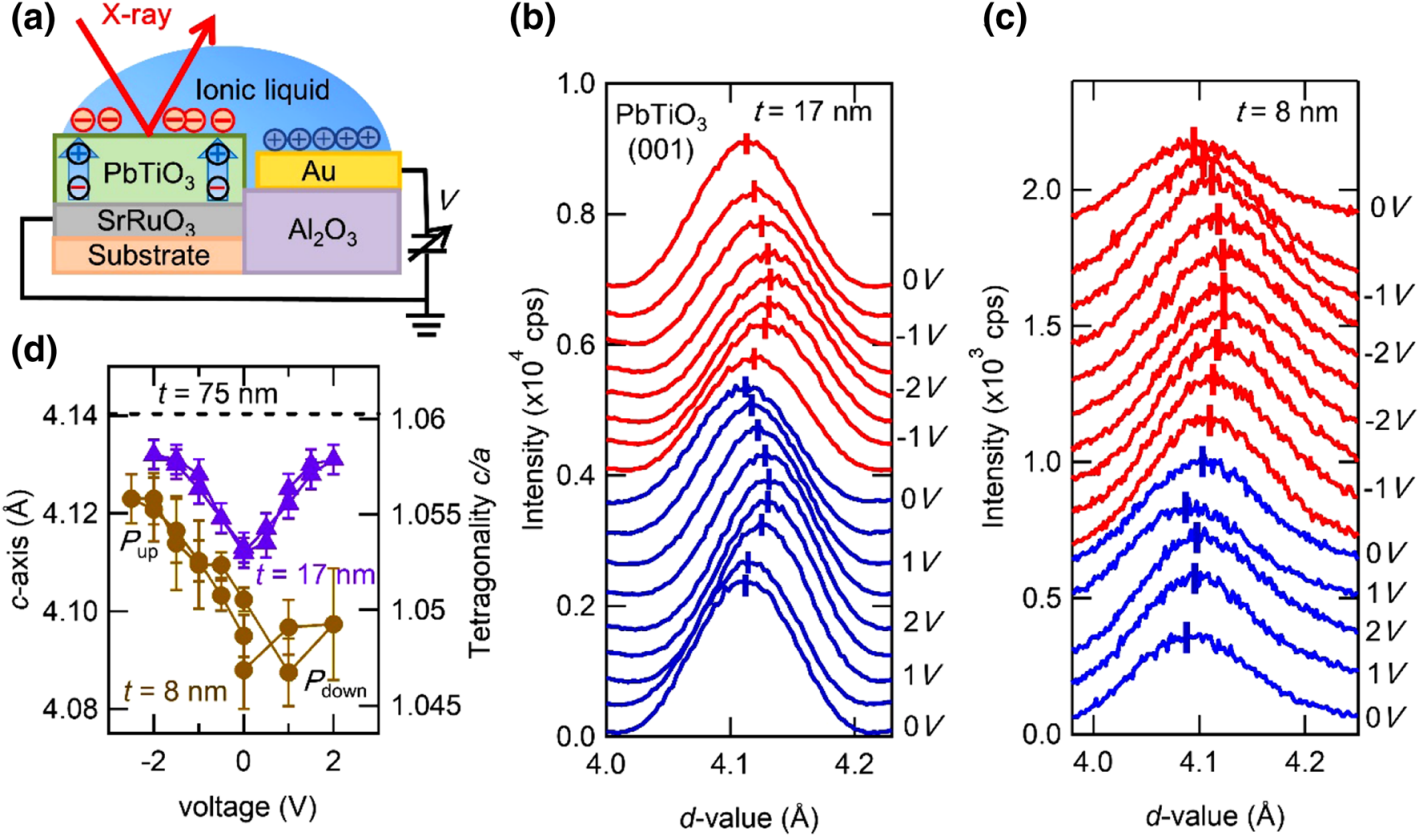
X-ray diffraction measurement under external electric field. (**a**) Schematic diagram of the device fabricated for in-situ X-ray diffraction measurement. Evolution of PbTiO_3_ (001) diffraction peaks in (**b**) 17 and (**c**) 8 nm-thick films under various applied voltages. Voltages are applied from 0 V → positive → negative → 0 V. (**d**) The measured *c*-axis lattice constant of PbTiO_3_ films as a function of the applied voltage. Positive and negative voltages stabilize downward (*P*_down_ state) and upward (*P*_up_ state) polarization, respectively.

Seen from Fig. 4a, b, we observe the trend that the small tetragonality shows small polarization and this may partially come from the depolarization field. Therefore, it is interesting to examine whether or not the reduced tetragonality in thinner films reverts to that in thicker films under an applied external electric field to cancel out the depolarization field. To examine this assumption, we performed *in-situ* X-ray measurements^34^ as shown in Fig. 5a. We measured *c*-axis lattice constant at various voltages. Figure 5b, c show the peak shift of PbTiO_3_ (001) diffraction under each external voltage for 17 and 8 nm-thick films, respectively. The *c*-axis elongates under the both positive and negative voltage, indicating the recovery of the tetragonality. The elongation of *c*-axis as a function of the external voltage is summarized in Fig. 5d, where the *c*-axis lattice constant of 75 nm-thick film at 0 V is indicated as the dotted line (see Supplementary Note 3 for the detail method to estimate *c*-axis lattice constant and error bar). The asymmetric *c*-axis elongation to applied voltage for 8 nm-thick film may be caused by an internal field such as built-in potential. Although the *c*-axis expands under an external bias especially in a negative voltage direction, the *c*-axis is almost saturated before it reaches the value for the 75 nm-thick film. This indicates that in addition to the depolarization field, unrevealed factors are acting on and destabilizing the ferroelectricity of ultrathin films. One possibility is surface effect caused by inhomogeneous polarization distribution near the surface layer^35^. This issue remains as a future study.

In conclusion, we have investigated the thickness dependence of the ferroelectricity in PbTiO_3_ films such as coercive field, lattice tetragonality and remnant polarization with employing the EDL gating technique. This technique enables us to observe distinct remnant polarization down to 2.6 nm-thick film and to clarify the evolution of remnant polarization with thickness. The depolarization field induces not only the reduction of spontaneous polarization but also the polarization relaxation, resulting in the small remnant polarization ~ 20 μC cm^−2^ for the 2.6 nm-thick film. The polarization control with the EDL gating has also clarified the lattice deformation of PbTiO_3_ films under an external voltage. Imperfect recovery of ferroelectricity is observed even though an external electric field is supposed to cancel out the depolarization field, which implies unrevealed factors destabilizing ferroelectricity in ultrathin films. Our findings and techniques will pave the way for a comprehensive understanding of ultrathin ferroelectric films, enabling the evaluation of unconventional ferroelectric materials, which are often suffer from high leakage current.

## Methods

### Film growth and characterization

PbTiO_3_/SrRuO_3_ heterostructures were grown on (001) oriented SrTiO_3_ substrates by a standard pulsed laser deposition (Pascal Co. Ltd.). The detail growth condition is described in ref^20^. X-ray diffraction measurement was performed by a Rigaku SmartLab to investigate tetragonality *c/a* as a function of film thickness. Out of plane lattice constant was calculated from PbTiO_3_ (003) diffraction peak. We also confirmed PbTiO_3_ is coherently grown on SrTiO_3_ substrates (*a* = 3.905 Å) from the reciprocal space mapping, enabling us to estimate tetragonality. Film thicknesses of PbTiO_3_ were determined by X-ray reflectivity method. The domain structure of PbTiO_3_ films was confirmed by a MFP-3D atomic force microscopy (OXFORD INSTRUMENTS) operating in the dual resonance tracking (DART) mode^36^. In DART mode, modulation voltage composed of two sinusoidal waves with frequencies of *f*_1_ and *f*_2_ drive cantilevers to track a shift of tip-sample contact resonance. Two lock-in amplifiers detect amplitude (*A*_1_, *A*_2_) and phase (*φ*_1_, *φ*_2_) signals from *f*_1_ and *f*_2_. We adopted either (*A*_1_, *φ*_1_) or (*A*_2_, *φ*_2_) for PFM images. We focus on the relative “phase difference” and “amplitude difference” since quantitative discussion is difficult in DART mode. The further details is described in ref^36^. We shifted phase values for regions out of poled areas to become zero. Hard cantilevers (spring constant *k* = 7 N/m) were used to reduce electrostatic contribution to the PFM signal^37^.

### Polarization measurement with employing an electric double layer gating

In order to reverse and evaluate polarization, we employed IL, *N*,*N*-diethyl-*N*-methyl-*N*-(2-methoxyethyl) ammonium bis (trifluoromethyl-sulfonyl) imide (DEME-TFSI), in which an electrode of Pt coil was immersed (see Fig. 2a). The IL was stored in a vacuum hot plate at 90 °C to remove residual water. Before the measurement, we dropped the IL on the sample surface and transferred it immediately to a vacuum cryostat with a back pressure of 1 × 10^−5^ Torr. A device area where the IL contacts on the sample surface is around 3 × 1.5 mm^2^. The measurement was performed at room temperature. For the polarization measurement, we adopted a positive-up negative-down method to accurately determine the remnant polarization. The details are given in ref^16^.

### In-situ XRD measurement

The sample was set in a homemade vacuum cell with a highly X-ray transparent plastic window, which was evacuated and kept below 3 Torr. Several lead wires are introduced to the cell making electrical contacts. In order to prevent a top electrode from blocking off the X-ray path, we adopted a side gate structure as shown in Fig. 5a. We used 8 and 17 nm-thick sample with the size of 4 × 2 mm^2^. For the 8 nm-thick sample, we fabricated PbTiO_3_/LaNiO_3_/SrTiO_3_ heterostructures to avoid the peak overlapping of PbTiO_3_ (001) and SrRuO_3_ (001) diffraction peaks. We measured the PbTiO_3_ (001) diffraction peak at various applied voltages.

## Supplementary information

Supplementary information.
