# Supplementary material for: Evolution of ferroelectricity in ultrathin PbTiO3 films as revealed by electric double layer gating
Source: Sci Rep. 2020 Jul 2;10:10864. doi: 10.1038/s41598-020-67580-8 (PMC7331690; doi:10.1038/s41598-020-67580-8)
Supplement: Supplementary file 1 — Supplementary information. [file 41598_2020_67580_MOESM1_ESM.pdf]

Evolution of ferroelectricity in ultrathin $PbTiO_3$ films as revealed by electric double layer gating

Ryutaro Nishino[1,*], Takahiro C. Fujita[1], Fumitaka Kagawa[1,2], Masashi Kawasaki[1,2]

[1]Department of Applied Physics and Quantum-Phase Electronics Center, University of Tokyo, Bunkyo, Tokyo 113-8656, Japan

[2]RIKEN Center for Emergent Matter Science (CMES), Wako, Saitama 351-0198, Japan

* Corresponding Author: Nishino@kwsk.t.u-tokyo.ac.jp

Supplementary Information

Supplementary Note 1 | Phase and amplitude image of piezoresponse force microscope for 5.8 nm and 2.6 nm-thick $PbTiO_3$ films

Figure S1 shows phase and amplitude images of the piezoresponse after negative and positive biases (V_{pole}) were applied in upper and lower rectangles, respectively. Line profile between as-grown and poled regions are also shown. We shifted phase values for regions out of poled areas to become zero. For 5.8 nm-thick sample, the PFM phase and amplitude of the upper rectangle shows the same contrast to that of the as-grown region (Figure S1(a)), indicating that the 5.8 nm-thick $PbTiO_3$ thin film is monodomain with upward polarization at as-grown region. For 2.6 nm-thick sample, one positon shows the similar phase and amplitude contrast to 5.8 nm-thick sample (Figure S2(b)), indicating monodomain with upward polarization at as-grown region. In Fig. S3(c) and (d), on the other hand, phase and amplitude at as-grown regions take values betweem upward and downward polarization regions because piezoresponse is partially canceled out when a tip is close to a domain boundary. These results indicate that the presense of upward and downward polarization domains at as-grown region. Such behavior is also reported in previous literature[1].

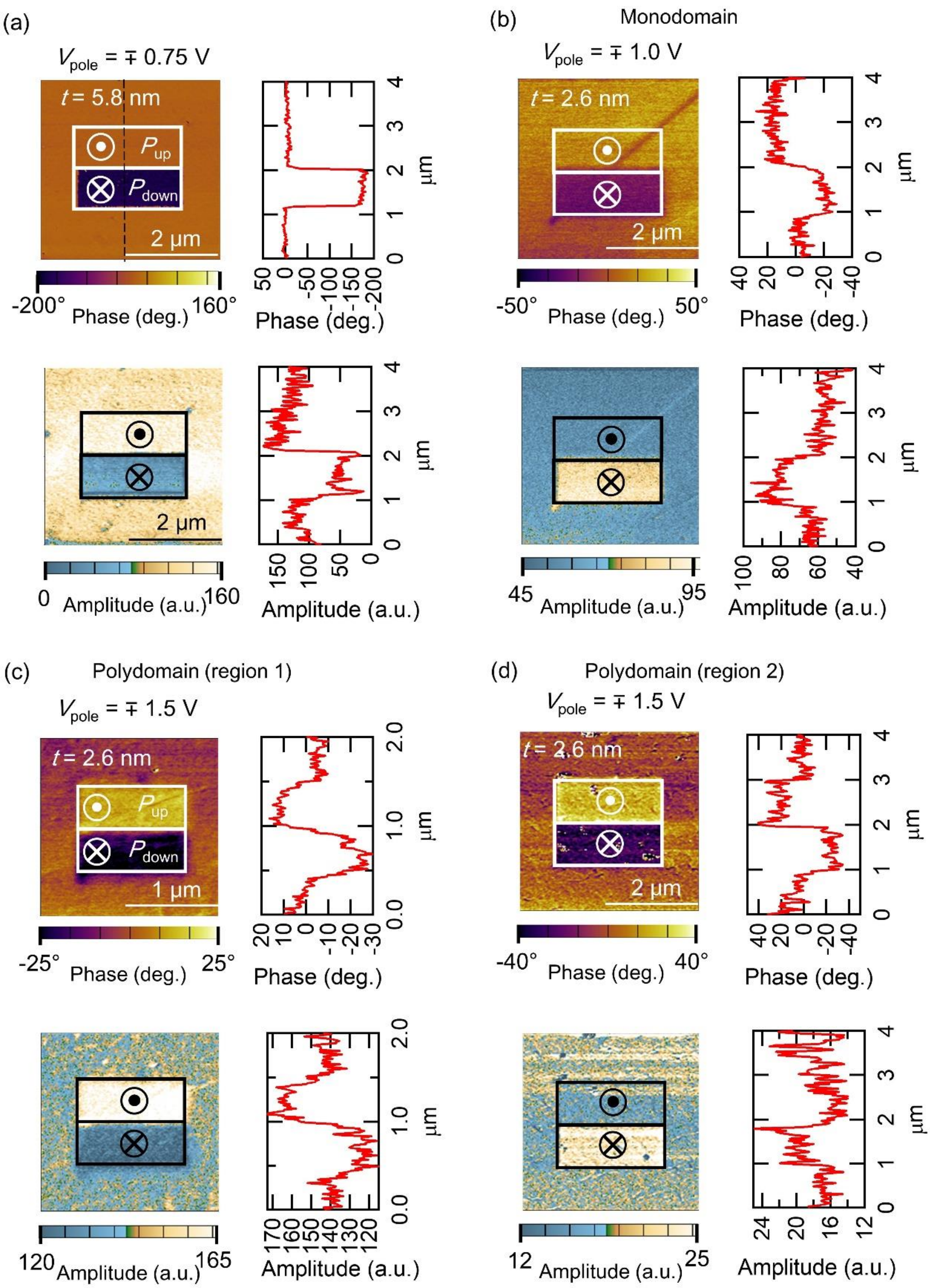

Figure S1. PFM images for $PbTiO_3$ films. Phase (top) and amplitude (bottom) images after negative and positive biases (V_{pole}) were applied in upper and lower rectangles, respectively. (a) 5.8 nm-thick film (b) 2.6 nm-thick film with monodomain state (c) 2.6 nm-thick with polydomain state (d) 2.6 nm-thick with polydomain state at different position.

Supplementary Note 2 | Kelvin probe force microscopy measurement for 2.6 nm-thick $PbTiO_3$ film

Figure S2(a) shows the surface potential image and line profile for 2.6 nm-thick $PbTiO_3$ film after negative and positive tip biases were applied to polarize the upper and lower rectangles, respectively. The surface potential of the upper rectangle region is negative while that of lower rectangle region is positive. The result reveals that the surface potential of the poled areas has an opposite sign to the remnant polarization, indicating that remnant polarization is over screened by the injected surface charges as shown in Fig. S2(b), being consistent with previous reports[2,3]. Figure S2(c) shows the time evolution of surface potential at the poled areas. The potential decay is slow and stable up to 16 h. We also scanned the poled areas with a grounded tip in contact mode to release the surface charges two hours after the poling. However, the surface potential does not show significantly change. This result indicates that the injected surface charges are trapped by the attractive force from remnant polarization.

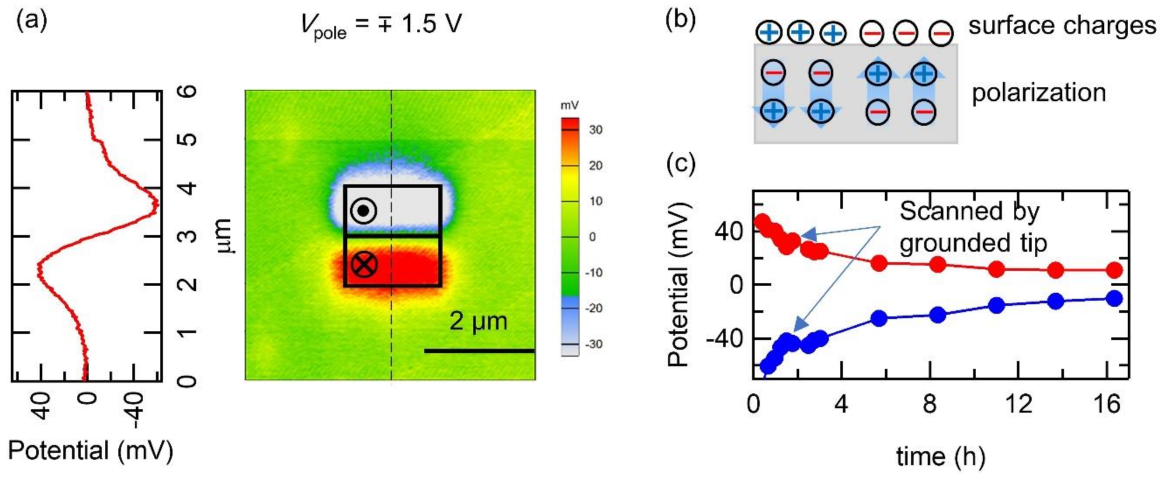

Figure S2. Kelvin probe force microscopy image for $PbTiO_3$ film (a) Surface potential image and line profile for 2.6 nm-thick $PbTiO_3$ film. Before the measurement, negative and positive biases (V_{pole}) were applied in upper and lower rectangles to induce upward and downward polarization, respectively. (b) Schematic of screening of remnant polarization by injected surface charges (c) Time evolution of surface potential at poled regions

Supplementary Note 3 | Peak assignment and error bar estimation for the in-situ x-ray diffraction measurements.

We used Multi-peak Fitting 2 package implanted in Igor Pro 6.35A (WaveMetrics) to determine peak positions from each in-situ X-ray diffraction measurement (in-situ XRD). We assumed Gaussian function for peak fitting. Figure S3(a) shows the fitting result of $PbTiO_3$ (001) diffraction peak for 17 nm-thick at 0 V. The diffraction peak is symmetric and the raw data is well fitted by single Gaussian function. In the in-situ XRD for 17 nm-thick film, we defined the resolution of our X-ray diffractometer (± 0.004 Å) as measurement errors. On the other hand, the diffraction peak for 8 nm-thick film at 0 V shows the asymmetric structure and the raw data is not well fitted by single Gaussian function as shown in Fig. S3(b). To obtain better fitting result, we assumed two Gaussian functions (peak 1 and peak 2) as shown in Fig. S3(c). The data is well fitted and we used peak 1 to estimate *c*-axis lattice constant of $PbTiO_3$. Two fitting methods (single or multi-peak fitting) show diffrent *c*-axis lattice constants. The difference is depends on applied voltage but typical value is around 0.008 Å, which is larger than the error caused by the resolution of our X-ray diffractometer. Therefore, we adopted the error caused by fitting methods as error bar for 8 nm-thick film in Fig. 5(d). The reason for the asymmetric peak structure is not clear. One possibility is a Laue fringe of a bottom electrode. For 8 nm-thick film, the diffraction peak of the bottom electrode is not completely ignored. Although we used $LaNiO_3$ films as a bottom electrode to avoid the peak overlapping of $PbTiO_3$ (001) and $SrRuO_3$ (001) diffraction peaks, a Laue fringe can be overlapped with $PbTiO_3$ peak or a Laue fringe of $PbTiO_3$ peak, which may induce some asymmetric structure.

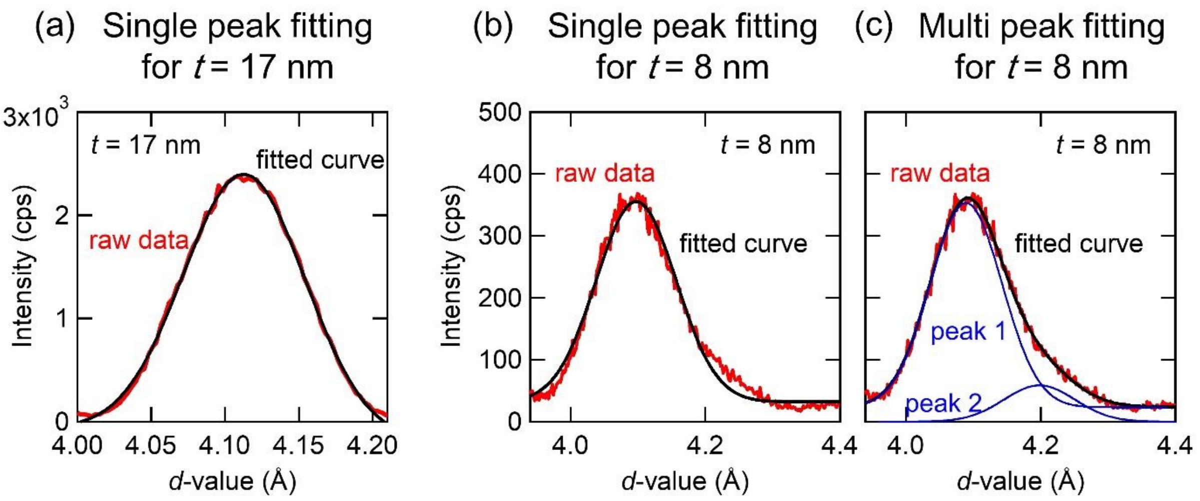

Figure S3. In-situ x-ray diffraction around $PbTiO_3$ (001) peaks at 0 V. Measured diffraction peaks (red) and fitted curves (black) for (a) 17 nm-thick film and (b) 8 nm-thick film. (c) Multi-peak fitting result for 8 nm-thick film. Two Gaussian peaks (peak 1 and peak 2) are assumed for fitting. Black fitted curve is the summation of peak 1 and peak 2.

Supplementary References

1. Lichtensteiger, C., Weymann, C., Fernandez-Pena, S., Paruch, P. & Triscone, J.-M. Built-in voltage in thin ferroelectric $PbTiO_3$ films: The effect of electrostatic boundary conditions. *New J. Phys.* **18**, 043030 (2016).
2. Chen, X. Q. *et al.* Surface potential of ferroelectric thin films investigated by scanning probe microscopy. *J. Vac. Sci. Technol. B Microelectron. Nanom. Struct.* **17**, 1930 (1999).
3. Kim, Y. *et al.* Origin of surface potential change during ferroelectric switching in epitaxial PbTiO3 thin films studied by scanning force microscopy. *Appl. Phys. Lett.* **94**, 032907 (2009).
